# Evaluation of efficacy of various immunochromatographic rapid tests for dengue diagnosis

**DOI:** 10.12669/pjms.301.4173

**Published:** 2014

**Affiliations:** 1 Dr. Arshi Naz, PhD, Research Fellow and Scientific Manager of Laboratory & Blood Bank Services,; 2 Danish Zahid, B.Sc, Scientific officer, National Institute of Blood Diseases & Bone Marrow Transplantation, ST 2/A Block 17 Gulshan-e-Iqbal KDA Scheme 24, Karachi, Pakistan.; 3 Dr. Samina Naz Murky, PhD, Assistant Professor, National Institute of Blood Diseases & Bone Marrow Transplantation, ST 2/A Block 17 Gulshan-e-Iqbal KDA Scheme 24, Karachi, Pakistan.; 4 Dr. Muhammad Nadeem, FCPS (Hematology), Consultant Hematologist and Head of Laboratory & Blood Bank Services, National Institute of Blood Diseases & Bone Marrow Transplantation, ST 2/A Block 17 Gulshan-e-Iqbal KDA Scheme 24, Karachi, Pakistan.; 5 Dr. Bijon Kumar Sil, Ph. D. Consultant Research and Development, MP Biomedicals Asia Pacific Pte Ltd, 2 Pioneer Place, Singapore, Sin-627885.; 6 Dr. Tahir Sultan Shamsi, FRCPath, Consultant Hematologist and Transplant Physician, National Institute of Blood Diseases & Bone Marrow Transplantation, ST 2/A Block 17 Gulshan-e-Iqbal KDA Scheme 24, Karachi, Pakistan.

**Keywords:** Immunochromatographic rapid test, Enzyme Linked Immuno Sorbent Assay (ELISAs), anti-dengue IgA

## Abstract

***Objective:*** The immunochromatographic rapid tests facilitate the early diagnosis of dengue by providing evidence of the presence of virus specific proteins (antigens/ antibody) in human blood. Many products for rapid dengue diagnosis are available in the market; the performance of few selected products was evaluated and compared with enzyme linked immuno sorbent assays (ELISA).

***Methods:*** Sera from a large number of patients (n=184) admitted to National Institute of Blood Diseases & Bone Marrow Transplantation (NIBD) were used to determine the efficiency of non-structural (NS) 1, IgA, IgG and IgM based rapid test devices for dengue diagnosis.

***Results:*** The dengue NS1 antigen based device was least efficient while among the antibody based devices the dengue IgA rapid test (RDT) was comparatively better (specificity: 80.95%; sensitivity: 85.21%). This device could detect both primary and secondary dengue infection and was found to be the most sensitive device at all point of sample collection.

***Conclusion:*** The dengue IgA RDT could be a cost effective and efficient rapid test device for timely dengue diagnosis at all levels of healthcare settings.

## INTRODUCTION

In the past few decades dengue has become a global health problem infecting millions of individuals annually.^1^^-^^3^ Since its first report in 1994 from the metropolitan city of Karachi dengue virus infection has become one of the expected causes of morbidity and mortality in Pakistan.^4^^,^^5^ All four serotypes of dengue virus (DENV-1 to DENV-4) are currently circulating and now become endemic in Pakistan with highest incidences reported during post monsoon period each year.^6^ A major outbreak of dengue virus infection in Pakistan was recorded during 2006.^7^ Clinically apparent disease due to dengue virus varies in severity from mild undifferentiated fever through to more severe forms such asd fever (DF) and dengue hemorrhagic fever (DHF). DHF is a vasculopathy characterized by capillary leakage and haematological deregulation; may result in fatal outcomes.^8^ An early and accurate diagnosis of dengue is important which assist not only in patient management by directing clinical attention but also in initiating community scale preventive measures for vector control to alleviate further dengue transmission. Commercial ELISA tests as well as immunochromatographic tests (RDT) that detect anti-dengue antibodies and the dengue NS1 antigen in plasma/sera have provided a new avenue for diagnosing dengue.^9^^,^^10^ In addition to this, the inexpensive and accurate dengue RDTs may also be useful in dengue epidemiological studies in resource limited countries. The primary and secondary dengue infections are diagnosed by the detection of either IgM or IgG or both in patient’s serum.^8^ These anti-dengue antibodies appear late in serum i.e. after 5-6 days of infection so NS 1 protein is detected in routine diagnosis during this period. The NS1 antigen appears in high concentration during the first few days of illness and it stays in serum after the infection subsides i.e. up to 14 days.^10^^,^^11^ Beside viral antigen detection a newer approach to dengue diagnosis is the use of anti-dengue IgA based RDT devices.^12^ IgA is the principal secretory antibody and human serum contains very low concentration of IgA1. During secondary dengue infection considerable increase in serum anti-dengue IgA is observed. The anti-dengue IgA is documented as the first antibody to appear in case of secondary dengue infection prior to IgG and IgM production while in case of primary infection it is produced after the anti-dengue IgM antibody due to class switching.^13^

In Karachi, most of the clinical diagnostic facilities are using commercial immunochromatographic rapid tests (RDT) from international manufacturers relying only on the supporting literature on the efficiency of these devices. No local data on the efficacy and sensitivity of most of these tests is documented.^14^ In this report three commercially available rapid diagnostic test devices were compared. Serological tests for dengue NS1 antigen, dengue IgA, dengue IgG/ IgM antibodies were carried out to demonstrate the potential application of these kits in early laboratory diagnosis of dengue infection with the special emphasis on utility of dengue IgA RDT as one of the new dengue diagnostic markers.^12^ The results were also compared with more specific and sensitive gold standard methods of dengue diagnosis i.e. IgG and IgM capture ELISAs.

## METHODS

***Study design: ***A panel of 184 dengue disease suspected human serum samples was used for the evaluation of efficacy of dengue immunochromatographic tests (RDT).^8^ The study was conducted during the period of August to December 2010 at National Institute of Blood Diseases (NIBD). A signed informed consent form and detailed questionnaire were obtained from patients involved in this study following institutional review board (IRB) policies. Age range of the study population was 2-65 years.

A probable dengue case may be a patient with presentation of defined clinical symptoms and history of acute febrile illness of 2–7 days with or without haemorrohage.^8^ In the present study any sample positive for either dengue specific-antigen (NS1) or antibodies (IgM or IgG or IgA) by RDT screening was considered as positive dengue case. All samples were characterized using dengue acute diagnostics: NS1 Ag Rapid Test (Panbio, Australia), ASSURE^® ^dengue IgA Rapid Test (MP Diagnostics, USA) and dengue Duo Cassette (Panbio, Australia). The efficiencies of all these devices were evaluated by comparing with dengue IgM capture ELISA (Dia. Pro, Italy) and dengue IgG capture ELISA (Dia. Pro, Italy). Complete blood count (CBC) was performed; after proper mixing by automated hematological analyzer; Sysmex XE-2100 (Sysmex Kobe, Japan); to evaluate the clinical parameters of dengue diagnosis (Platelets count, PCV, TLC etc).

***Dengue Antibodies ELISAs: ***Dengue IgM-Cap ELISA and IgG-Cap ELISA were used to detect dengue antibodies as per manufacturer’s instruction. Briefly, 100 µl/well of patient sera (diluted 1:100) and controls were added to the assay plate coated with either anti-human IgM or anti-human IgG to capture the IgM or IgG. Both plates were incubated for one hour at 37C. Then the plates were washed and 100 µl/well of enzyme conjugate was transferred to the assay plates except the blank wells. After 10 minutes, the reaction was stopped by addition of 100 µl/well 1 M phosphoric acid. The OD values were determined at 450 nm. A sample was defined as positive when ratio of sample absorbance and calculated cut-off value was > 1.

***Dengue NS1 Ag Rapid Test: ***Dengue NS1 Ag Rapid Test is a one-step chromatographic assay which uses murine monoclonal antibodies to capture dengue virus NS1 antigen in human serum or plasma and detected by NS1 antigen polyclonal antibodies conjugated with gold colloid. Assays were conducted and results were interpreted according to the instruction of manufacturers.

***Dengue IgA Rapid Test: ***ASSURE^® ^Dengue IgA Rapid Test (WO 2009/139725 ) is a reverse flow technique (US Patent-6316205) immunochromatographic rapid test for the qualitative detection of anti-dengue IgA from patient’s biological samples (serum, whole blood or plasma). In this platform, test sample migrates upwards from the sample well and eventually form antibody-antigen complexes with dengue virus antigens in the test window. The bound antibody-antigen complexes is subsequently detected by goat anti-human IgA gold conjugate carried by chase buffer that flows downwards giving a pink-purplish color line in the test window. Control line which contains protein L captures human IgA from patient’s sample and binds with the anti-human IgA-gold conjugate, resulting in pink-purplish color line in the control window. The appearance of control line indicates the proper addition and migration of serum sample and chase buffer. The test was performed as per manufacturer’s instruction for use (IFU) and results were interpreted according to the intensity scale provided with the kit box without knowledge of the results of other tests.

***Dengue Duo IgM/IgG Cassette: ***Dengue Duo Cassette is a lateral flow based immunochromatographic rapid test for the qualitative detection of anti-dengue IgM and IgG from patient’s biological samples (serum, whole blood or plasma). The device is designed to detect IgM antibodies to dengue, as well as elevated IgG titers that are indicative of a secondary infection. The rapid tests were read and interpreted according to the manufacturers' instructions. The positivity of individual marker were calculated separately (IgM or IgG) as well as in combination (IgM and IgG).

***Statistical analysis: ***The quantitative variables were expressed as mean, standard deviation, confidence interval and minimum and maximum range. The qualitative variables were mentioned as percentages. The sensitivity and specificity of immunochromatographic tests were analyzed by 2x2 table using MedCalc statistical software, version-11.5 (Broehstraat 52, B-9030 Mariakerke, Belgium). Kappa value was used to test the efficiency and reliability of the rapid devices.

## RESULTS

***Characteristics of the study population: ***The characteristics of the study population (n = 184 cases) that contributed acute sera to the test panel is shown in Table-I. The median duration of illness prior to the test plasma sample being collected was 5 days (range: 2-17). There were 42 patients with no viral antigen (NS1) or serological evidence of acute or recent dengue infection in collected serum specimens. Of 142 dengue positive samples, 97 samples were considered as secondary infection (positive for either IgM and Ig G or positive for IgG by the standard capture ELISAs) and 45 samples were dengue primary infection (positive with IgM capture ELISA and negative with IgG capture ELISA).

***Sensitivity and specificity of dengue immunochromatographic tests versus reference*** ***dengue tests*** **:** The sensitivities and specificities of three RDTs against the reference tests i.e. dengue IgM-cap ELISA and IgG-cap ELISA are presented in Table-II. The dengue NS1 antigen based RDT showed 64.08% (91/142) sensitivity compared to 72.54% (103/142) by Den IgM/IgG RT. The individual dengue diagnostic marker of Panbio IgM/IgG RDT showed 63.38% (IgM) and 48.59% (IgG) respectively. The dengue IgA RDT showed higher level of performance over NS1 and IgM/IgG RDT and was found to 85.21% (121/142). In terms of specificity dengue NS1 showed 100% accuracy compared to 69.05% (29/42) and 80.95% (34/45) by dengue IgM/IgG RDT and dengue IgA RDT respectively.

***Immunochromatographic tests sensitivity in primary or secondary infection: ***The detection of both dengue primary and secondary infected patients by dengue IgA RT was significantly higher than Panbio IgM/IgG Duo RDT and is presented in Table-III. The primary dengue infection was confirmed by positive anti-dengue IgM and negative anti-dengue IgG-cap by reference ELISAs. The dengue IgA RDT detected 91.11% (41/45) dengue primary infected cases compared to 75.56% (34/45) by Dengue IgM/IgG Duo RDT. Like-wise dengue IgA RDT detected 82.47% cases of dengue secondary infection compared to dengue Duo IgM/IgG RDT (71.13%). Moreover, the dengue secondary marker of IgM/IgG RDT (IgG) showed lowest level of detection of dengue secondary infection (51.55%).

***Sensitivity of Immunochromatographic tests by day of illness: ***The sensitivity of three RDTs was also evaluated based on the duration of onset of dengue fever, categorized into 6 groups (Table-IV). The dengue IgA RDT showed significantly higher level of detection of dengue cases at all points of collection of samples (day 1-13) compared to dengue Duo IgM/IgG RDT and dengue NS1 RDT.

## DISCUSSION

Serological assays from a single serum specimen using RDT device, in many instances, provide presumptive diagnosis of dengue infections.^13^^,^^15^ The commonly occurring infection in Pakistan is generally dengue fever but rare cases of DHF particularly DSS mostly result in mortality due to improper diagnosis or false negative results on most of the RDT devices.^16^ There remains a need for more efficient sero-diagnostic device. Therefore, in this study, we have included acute dengue suspected samples in the comparative evaluation of routine dengue- NS1, IgM, IgG and a new IgA RDT with WHO recommended IgM and IgG capture ELISAs.^8^

**Table-I:** Demographic and clinical features of **dengue suspected cases**

*Variables*	*Confirmed dengue cases*	*Non-dengue febrile illness*
Male	52.17% (n=96)	15.76% (n=29)
Female	25.00% (n=46)	7.00% (n=13)
Age	28.06±14 (2-65 yrs)	27.42±15.2 (6-65 yrs)
Platelets	80.76±84.8 (2-421 x 10^9^/L)	70.13±60.22 (11-254 x 10^9^/L)
PCV	38.97±6.69 (19-52%)	39±5.89 (28-47%)
TLC	5.32±3.09 (1.09-16.05 x 10^9^/L)	4.38±2 (0.66-9.37 x 10^9^/L)
Day of illness	5±2.1 (1-14 days)	5.63±3.4 (3-20 days)
Temperatures	101.97±0.87 (100-105ºF)	101.96±0.75 (101-104ºF)
Primary infection	68.31% (n=97)	-
Secondary infection	31.69% (n=45)	-

**Table-II:** Efficiency of rapid test devices

*Rapid Tests*	*Sensitivity * (95% CI)*	*Specificity* *(95% CI)*	*Positive Predictive Value (95% CI)*	*Negative Predictive Value (95% CI)*
Dengue-IgA RDT	85.21% (121/142) (78.29% to 90.61%)	80.95% (34/42)) (65.88% to 91.40%)	93.80% (88.15% to 97.28%)	61.82% (47.73% to 74.59%)	
Dengue-IgM RDT	63.38% (90/142) (54.89% to 71.30%)	76.19% (32/42) (60.55% to 87.95%)	90.00% (82.38% to 95.10%)	38.10% (27.71% to 49.34%)	
Dengue-IgG RDT	48.59% (69/142) (40.13% to 57.12)	76.19% (32/42) (60.55% to 87.95%)	87.34% (77.95% to 93.76%)	30.48% (21.87% to 40.22%)	
Dengue-IgM/IgG RDT	72.54% (103/142) (64.42% to 79.68%)	69.05% (29/42) (52.91% to 82.38%)	88.79% (81.60% to 93.90%)	42.65% (30.72% to 55.23%)	
Dengue-NS1 RDT	64.08% (91/142) (55.61% to 71.96%)	100% (42/42) (91.59% to 100.00%)	100% (96.03% to 100.00%)	45.16% (34.81% to 55.83%)	

*95% confidence Interval (**CI**)

**Table-III:** Comparative performance of anti-**dengue** antibody based **chromatographic rapid tests** against **dengue** reference ELISAs in primary and secondary infection

*Dengue Diagnostics*	*Sensitivity and Specificity*		
	*Primary infection (n=45)-95% CI* ^*^	*Secondary infection* (n=97)-95% CI*	*Total (%)* (n=97)-95% CI*
Dengue IgA RT	91.11 (41/45) (78.78% to 97.52%)	82.47% (80/97) (73.43% to 89.45%)	85.21% (121/142) (78.29% to 90.61%)
Dengue-IgM RT	60.67% (30/45) (51.05% to 80.00%)	61.86% (60/97) (51.43% to 71.53)	63.38% (90/142) (54.89% to 71.30%)
Dengue-IgG RT	42.22% (19/45) (27.66% to 57.85%)	51.55% (50/97) (41.18% to 61.82%)	48.59% (69/142) (40.13% to 57.12%)
Dengue-IgM/IgG RT	75.56% (34/45) (60.46% to 87.12%)	71.13% (69/97) (61.05% to 79.89%)	72.54% (103/142) (64.42% to 79.68%)

*95% confidence Interval (CI)

**Table-IV:** Detection of dengue infection at different stages of disease by various RDTs (n=71.19%/142).

*Days after onset of fever* *(no. of cases)*	*Total cases detected* *(%)*						
	**Dengue- IgA RDT**	*Dengue-IgM RDT*	*Dengue-IgG RDT*		*Dengue- IgM/IgG RDT*		*Dengue-NS1 RDT*
Day-1-3 (20)	90.00	60.00		35.00		70.00	75
Day-04 (38)	81.58	50.00		36.84		60.53	63.16
Day-05 (40)	80.00	70.00		65.00		82.50	55
Day-06 (20)	90.00	75.00		60.00		75.00	75
Day-07 (11)	90.91	63.64		27.27		63.64	90.91
Day>07 (13)	92.31	69.23		53.85		84.62	38.46
Total (n=142)	85.21%	63.38%		48.59%		72.54%	64.08%

The serum IgA levels are documented as another approach for early detection of dengue infection.^15^^,^^17^ IgA is the second most abundant antibody in human body. It is found in serum in both primary and secondary dengue infections. During primary infection it appears ~1 day after IgM; requires class switching by somatic hypermutation. Talarmin et al suggested anti-dengue IgA as a marker of recent dengue infection in dengue endemic areas.^18^ In acute secondary infection unlike IgG and IgM antibodies the anti-dengue IgA antibodies appear earlier in serum i.e. even prior to active viremia.^13^ Generally, dengue secondary infection is most likely to be associated with the more serious form of the disease, thus the detection of dengue secondary cases at the early stage of infection is of prime importance for proper medical attention to avoid fatal outcomes.^8^^,^^19^ The dengue IgA RDT used during this study was found to detect more cases of dengue secondary infection compared to IgG RDT Duo cassette Table-III. These observations are in line with a previous study on dengue IgA RDT.^12^ The specificity and sensitivity of dengue IgA RDT in earlier studies were quite similar to our findings 86.70% (85.21%) and 86.05% (80.95%) respectively. Additionally, the level of detection of infection at all stages of disease by IgA RDT was far better (negative predictive value: 61.82%; Table-II). Ahmed & coworkers validated the same IgA RT to be 99.4% sensitive and 99.2% specific with 100% detection of primary dengue cases of all serotypes compared with reverse transcriptase polymerase chain reaction.^20^

Furthermore, among the three tested RDT devices the NS-1 antigen based device was found least efficient with 35.91% (51/142) false negative results. Dengue NS-1 antigen can be found in patient’s serum until the production of anti-NS-1 IgG or in some instances a few days after defervescence. The variable levels of NS-1 antigen in different phases of infection might be due to the immune-complex formation of NS1 antigen with anti-dengue IgG resulting in less sensitive dengue detection in acute-phase secondary infection as reported previously.^21^

## CONCLUSION

We conclude that the serological testing for early diagnosis of dengue infections could be accomplished by using IgA based RDT at all heath care settings during any stage of infection cycle. Moreover, it partially overcomes the limitations associated with ELISAs like handling of single sample without delay.

## Notes

***Note: ***The following posters were also presented from this project”

- Comparison of 3 commercial rapid immunochromatographic devices for the diagnosis of acute Dengue virus in Karachi, Pakistan (8^th^ Annual Conference of Infectious Diseases in Islamabad, Pakistan, March 2011)
- Diagnosis of Dengue (53^rd^ annual National Student Research Forum in Texas, USA, April 2012)
